# Synergistic Effects of Dantrolene and Nimodipine on the Phenylephrine-Induced Contraction and ACh-Induced Relaxation in Aortic Rings from Diabetic Rats

**DOI:** 10.1155/2018/9790303

**Published:** 2018-04-19

**Authors:** Maria J. Crespo, Marie Roman, Jonathan Matias, Myrna Morales, Hector Torres, Jose Quidgley

**Affiliations:** ^1^Department of Physiology, University of Puerto Rico-School of Medicine, San Juan, PR, USA; ^2^Department of Anesthesiology, University of Puerto Rico-School of Medicine, San Juan, PR, USA

## Abstract

Diabetics have a higher risk of developing cerebral vasospasms (CVSP) than nondiabetics. The addition of the ryanodine receptor (RyR) blocker dantrolene to standard therapies reduces vasospasms in nondiabetics. Whether diabetics with CVSP also benefit from this drug, however, is unknown. We evaluated the effects of a 30 min incubation with dantrolene (50 *μ*M), nimodipine (50 nM), and both drugs in combination, on phenylephrine- (PHE-) induced contraction and on acetylcholine- (ACh-) induced relaxation in aortic rings from streptozotocin (STZ) diabetic rats. Age-matched, nondiabetic rats served as controls. The oxidative stress markers malondialdehyde (MDA) and 4-hydroxyalkenal (4-HAE) were also evaluated in the presence and absence of dantrolene and nimodipine. The combination of these two drugs acted synergistically to reduce the PHE-induced contraction by 80% in both diabetics and controls. In contrast, it increased the *E*_max_ value for ACh-induced relaxation (from 56.46 ± 5.14% to 96.21 ± 7.50%; *n* = 6, *P* < 0.05), and it decreased MDA + 4-HAE values in diabetic rats only. These results suggest that the combination of dantrolene and nimodipine benefits both diabetics and nondiabetics by decreasing arterial tone synergistically.

## 1. Introduction

Diabetic patients have a higher incidence of vascular complications and are more prone to developing cerebral vasospasms (CVSPs) than nondiabetics [1]. Indeed, CVSPs are responsible for the majority of diabetic morbidity and mortality [2]. Although the etiology of CVSPs has not been determined, it has been associated with increased vascular tone due to persistent elevation of intracellular Ca^2+^ in vascular smooth muscle (VSM) [2, 3], which may be secondary to (1) increased extracellular Ca^2+^entry into the cell [4] or to (2) increased release of this ion from the sarcoplasmic reticulum (SR), which is mediated by the ryanodine receptors (RyRs) [5]. Alterations in the homeostasis of multiple mediators, including nitric oxide, serotonin, and endothelin-1, have also been proposed to underlie the onset and development of CVSP [6].

Cardiovascular abnormalities in diabetes have been linked to increased oxidative stress and endothelial dysfunction [7–9]. Moreover, unlike in nondiabetics, alterations in the status of endothelial nitric oxide synthase (eNOS) and inducible nitric oxide synthase (iNOS) characterize diabetic animal models and patients [10–13] and may contribute to increased vascular tone. These findings suggest that the pathophysiology of CVSP differs between diabetics and nondiabetics. The current treatment for this condition is similar for these two groups of patients, however, and includes the use of nimodipine, nicardipine, and other voltage-dependent L-type Ca^2+^ channel antagonists [1]. Nevertheless, these drugs are not completely effective in reducing CVSPs because Ca^2+^ antagonists only block the influx of Ca^2+^ into the cytosol without interfering with the Ca^2+^ released from the SR. Therefore, cerebral infarction, neurological complications, and death rates remain elevated in patients, despite the use of Ca^2+^ antagonists [3].

Dantrolene is a peripherally acting skeletal muscle relaxant approved for the treatment of spasticity, malignant hyperthermia, and neuroleptic malignant syndrome. It depresses excitation-contraction coupling by inhibiting Ca^2+^ release from the SR throughout the blockade of the RyRs present in the VSM [14, 15]. Indeed, mRNAs from RyR1, RyR2, and RyR3 subtypes are expressed in the VSM of the thoracic aorta [15], the mesenteric arteries [15], the large cerebral arteries [16], and the cerebral microcirculation [5], indicating that the VSM from different sized vessels contains the three RyR isoforms. In nondiabetic patients, concomitant administration of the RyR blocker dantrolene with a Ca^2+^ channel antagonist improves vascular and neurologic outcomes because this combination reduces vasoconstriction more than either drug alone [17].

It is unknown, however, whether the combination of a Ca^2+^ channel blocker with dantrolene will also benefit diabetic patients because, in addition to increasing oxidative stress and inducing endothelial dysfunction, diabetes alters SR Ca^2+^ content, SR protein expression, RyR2 mRNA and RyR2 protein levels in a rat heart [18], and L-type Ca^2+^ channel regulation and expression in VSM [4]. Thus, it is crucial to determine if adding dantrolene to standard therapies with Ca^2+^ channel blockers is also beneficial to diabetic patients. In this study, we investigate the effects of dantrolene, either alone or in combination with nimodipine, on phenylephrine- (PHE-) induced contraction, and in the acetylcholine- (ACh-) induced relaxation of aortic rings from streptozocin- (STZ-) induced diabetic rats. In addition, in rabbits following traumatic spinal cord injury, dantrolene is known to decrease malondialdehyde (MDA) levels, an indicator of lipid peroxidation, and increase reduced glutathione (GSH) [14], while in spinal cord-injured rats, nimodipine provides protection against oxidative stress [19]. We also assess the effects of dantrolene and nimodipine on oxidative stress by evaluating malondialdehyde (MDA) and 4-hydroxyalkenal (4-HAE) levels in vascular homogenates in order to determine if the antioxidant effects of these drugs observed in spinal cord injury [14, 19] occur in the vasculature as well.

## 2. Drugs

Dantrolene, nimodipine, phenylephrine (PHE), sodium nitroprusside (SNP), and N^G^-nitro-L-arginine (L-NAME) were obtained from Sigma Chemical Co. (St. Louis, MO). Kits for lipid peroxidation were obtained from Percipio Biosciences (Burlingame, CA). The concentrations of nimodipine (50 nM) and dantrolene (50 *μ*M) were selected based on comparable studies in different vascular beds from rats [20–22].

## 3. Materials and Methods

### 3.1. Experimental Animal Model

Forty male Sprague-Dawley rats (Taconic Biosciences Inc., Germantown, NY), approximately four weeks of age, were divided into the diabetic group and the nondiabetic group, with each group containing 20 animals. To induce diabetes, the rats were fasted overnight and then injected IP with streptozotocin (STZ, 65 mg/kg) dissolved in a 0.1 M citrate buffer (pH 4.5). Nondiabetic animals, which were used as controls, were only injected (IP) with the citrate buffer solution. Hyperglycemia was verified 24 h after the STZ injection with a TRUEtrack blood glucose monitoring system (NIPRO Diagnostics, Fort Lauderdale, FL). Blood glucose levels were monitored once a week in all animals. All the experiments were performed at four weeks following diabetes induction. The diabetic rats never received insulin supplementation. All animals were housed in a temperature-controlled room on a 12 h light/dark cycle. Water and food (Harlan Rodent Diet, 18% protein) were provided ad libitum. All procedures involving the animals were approved by the Institutional Animal Care and Use Committee (protocol number 2590115) and adhered to the *Guide and Care for the Use of Laboratory Animals* published in 2011 by the National Institutes of Health (USA).

### 3.2. Tissue Preparation for Isometric Tension Studies

To evaluate endothelial-dependent relaxation, we followed the methods of Quidgley et al. [23]. Briefly, rats were anesthetized with a combination of ketamine (50 mg/kg, IP) and xylazine (4 mg/kg, IP). After full anesthesia, aortic rings of approximately 5 mm in length were obtained from the proximal segment of each aorta and used for the contraction and relaxation studies. The connective tissue adjacent to the aortic adventitia was carefully removed, avoiding damages to the smooth muscle and the endothelium. The entire preparation was mounted in a two-hook, 50 ml organ chamber (Radnoti Co., Monrovia, CA) and bathed in Krebs' bicarbonate solution (aerated with a mixture of 95% O_2_ and 5% CO_2_ at 37°C). The rings were suspended horizontally with a resting tension of 2.3 g and connected to a FT03C Grass transducer. Once the optimal tension was reached, the rings were subjected to a 1 h equilibration. The signal was analyzed with a data acquisition card (National Instruments, Austin, TX; PC-LPM-16/PnP), and changes in isometric tension were recorded with LabView software (National Instruments).

### 3.3. Measurement of Aortic Ring Contraction and Relaxation

To determine the effect of 50 *μ*M dantrolene, 50 nM nimodipine, and the combination of these drugs on the endothelial-dependent relaxation, aortic rings were precontracted with phenylephrine (PHE, 1.0 *μ*M), following the protocol described previously [23, 24]. When the maximal contraction reached a plateau, cumulative concentration-response curves (from 0.1 nM to 10 *μ*M) for ACh were generated. After the completion of the curves, the rings were washed and stabilized. Additional concentration-response curves were then performed after a 30 min incubation period with the drugs. In each experiment, the relaxation was expressed as a percentage of the relaxation relative to the maximal contraction induced by 1.0 *μ*M of PHE. The maximal relaxation achieved (*E*_max_) and the concentrations inducing 50% of maximal relaxation (EC_50_) for ACh were determined before and after incubation with dantrolene, nimodipine, and these drugs in combination through the mathematical analysis of the concentration-response curves, which were then compared. Sodium nitroprusside (SNP, 1.0 *μ*M) was also used to fully relax aortic rings after the completion of each protocol to assess endothelial-independent relaxation.

To evaluate the role of nitric oxide (NO) in ACh-induced relaxation after incubation with dantrolene, nimodipine, or these drugs in combination, the aortic rings were incubated with 1 mM N^G^-nitro-L-arginine (L-NAME), an inhibitor of NOS, concomitantly with the drugs. In these experiments, the rings were precontracted with 1.0 *μ*M of PHE, and a concentration-response curve for ACh was generated before and after incubation with L-NAME and each drug, either alone or in combination.

The effects of nimodipine, dantrolene, and its combination on the PHE-induced contraction were tested by analyzing the cumulative concentration-response curves generated by PHE (0.1 nM to 10.0 *μ*M) in aortic rings before and after incubation with the drugs. The maximal contraction (*E*_max_) and the concentration inducing 50% of maximal contraction (EC_50_) for PHE were also determined through the analysis of the concentration-response curves for each individual group.

### 3.4. Measurement of Malondialdehyde and 4-Hydroxyalkenal Levels

The effects of dantrolene and nimodipine on lipid peroxidation, a marker of oxidative stress, were evaluated following the methods of Quidgley et al. [23], by measuring vascular malondialdehyde (MDA) and 4-hydroxyalkenal (4-HAE) levels before and after a 30 min incubation period with dantrolene (50 *μ*M), nimodipine (50 nM), and these drugs in combination using a commercial kit (Bioxytech LPO-586; Oxis Research, Portland, OR). Briefly, homogenized tissues were centrifuged at 4°C for 10 min, and the supernatant was collected and stored at −80°C. N-Methyl-2-phenylindole and methanesulfonic acid were added to each sample and incubated at 45°C for 60 min. Samples were then centrifuged, and the supernatant was transferred to a cuvette. The absorbance of each sample was measured at 586 nm using a SpectraMax M3 Microplate Reader (Molecular Devices, Silicon Valley, CA). The MDA and 4-HAE concentrations of each sample were compared to the standard curve generated under the same conditions and were normalized by protein concentration, as determined by Bradford's Assay [25].

## 4. Statistical Analysis

Results are presented as the mean ± SEM using statistical software (GraphPad Prism 5.03, GraphPad Software Inc., San Diego, CA). Statistical comparisons between groups were performed using Student's *t*-test when comparing only two variables and the analysis of variance (ANOVA) when comparing more than two groups. The Student-Newman-Keuls test for post hoc analysis was used to further evaluate significant ANOVAs. Values were considered statistically significant at a *P* value less than 0.05.

## 5. Results

The general characteristics of the experimental animals are shown in Tables 1 and 2. Blood glucose levels in diabetic rats were higher than 400 mg/dl during the study period, ranging from 451.7 ± 41.5 mg/dl at 24 h following STZ administration to 445.4 ± 63.7 mg/dl after four weeks. In control animals, by contrast, glucose concentration remained within the normal range throughout the study, with a mean value of 143.5 ± 6.8 mg/dl. In addition, whereas body weight increased in both diabetic and control rats over the course of this study, it remained significantly lower in the diabetic rats at four weeks following diabetes induction (272.2 ± 24.8 g in diabetics versus 360.6 ± 5.9 g in controls; *n* = 20, *P* < 0.05).

**Figure 1:**
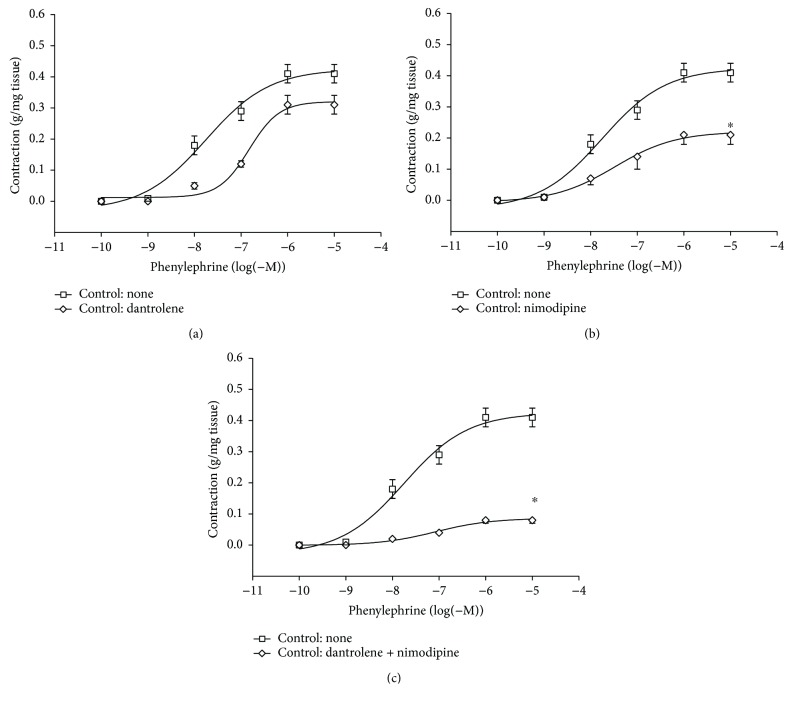
Cumulative concentration-response curves (from 0.1 nM to 10 *μ*M) for the phenylephrine- (PHE-) induced contraction of aortic rings from control rats (CT) only before and after a 30 min incubation period with 50 *μ*M dantrolene (a), with 50 nM nimodipine (b), and with both drugs in combination (c). The values shown are the means ± SEM of 6 to 9 animals per group. ^∗^*P* < 0.05, when comparing *E*_max_ before and after incubation with nimodipine and with dantrolene and nimodipine in combination.

**Figure 2:**
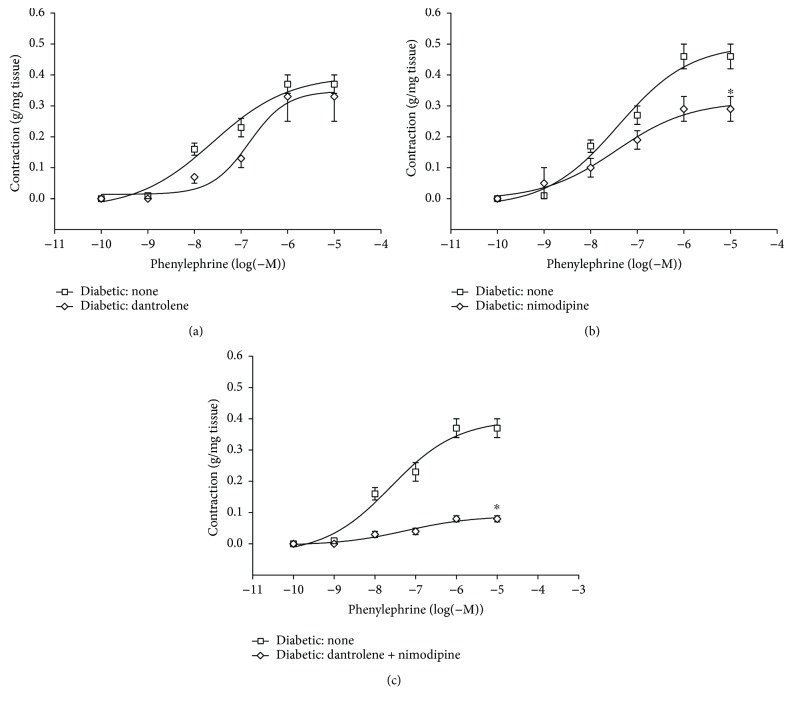
Cumulative concentration-response curves (from 0.1 nM to 10 *μ*M) for the phenylephrine- (PHE-) induced contraction of aortic rings from STZ diabetic rats only before and after a 30 min incubation period with 50 *μ*M dantrolene (a), with 50 nM nimodipine (b), and with both drugs in combination (c). The values shown are the means ± SEM of 6 to 9 animals per group. ^∗^*P* < 0.05, when comparing *E*_max_ before and after incubation with nimodipine and with dantrolene and nimodipine in combination.

Cumulative concentration-response curves for the PHE-induced contraction of aortic rings are shown in Figure 1 for control rats and in Figure 2 for diabetic rats. A 30 min incubation period with 50 *μ*M dantrolene increased the EC_50_ value for PHE from 43.2 ± 8.11 to 118.72 ± 17.27 nM (*P* < 0.05) in controls (Figure 1(a), Table 3) and from 50.83 ± 7.78 to 118.73 ± 16.47 nM (*P* < 0.05) in diabetics (Figure 2(a), Table 3). Incubation with 50 nM nimodipine reduced *E*_max_ in controls (Figure 1(b)) by 53% (*P* < 0.05; *n* = 9) and by 37% (*P* < 0.05; *n* = 9) in diabetics (Figure 2(b)). The combination of dantrolene and nimodipine was equally effective in reducing the PHE-induced contraction by about 80% (*P* < 0.05; *n* = 9) in both controls (Figure 1(c)) and diabetics (Figure 2(c)).

**Figure 3:**
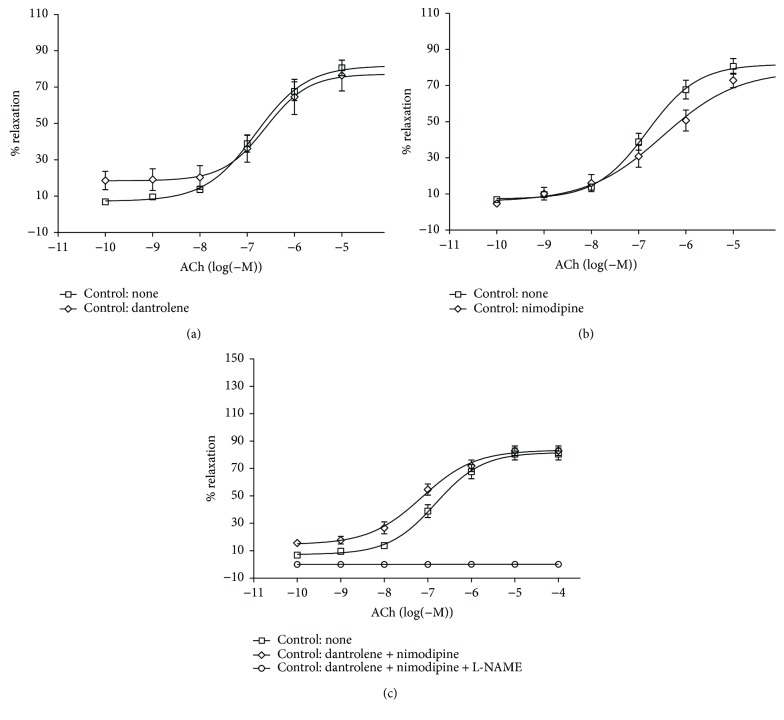
Cumulative concentration-response curves for the acetylcholine- (ACh-) induced relaxation of aortic rings from control rats (CT) only after a 30 min incubation period with 50 *μ*M dantrolene (a), with 50 nM nimodipine (b), and with both drugs in combination (c). Aortic rings were precontracted with 0.1 *μ*M phenylephrine (PHE) before the addition of cumulative concentrations of ACh. The values shown are the means ± SEM of 6 to 9 animals per group. Note that the addition of 1 mM L-NAME to the incubation bath inhibited the ACh-induced relaxation of aortic rings from dantrolene and dantrolene + nimodipine-treated control rats.

**Figure 4:**
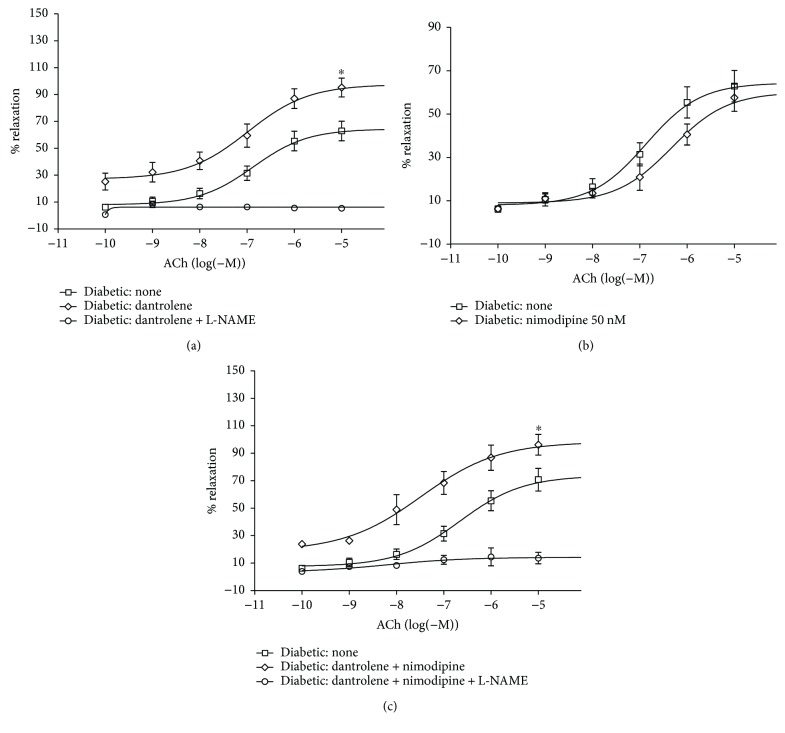
Cumulative concentration-response curves for the acetylcholine- (ACh-) induced relaxation of aortic rings from STZ diabetic rats after a 30 min incubation period with 50 *μ*M dantrolene (a), with 50 nM nimodipine (b), and with both drugs in combination (c). Aortic rings were precontracted with 0.1 *μ*M phenylephrine (PHE) before the addition of cumulative concentrations of ACh. The values shown are the means ± SEM of 6 to 9 animals per group. Note that the addition of 1 mM L-NAME to the incubation bath inhibited the ACh-induced relaxation of aortic rings from dantrolene and dantrolene + nimodipine-treated diabetic rats. ^∗^*P* < 0.05, when comparing *E*_max_ before and after incubation with dantrolene and with dantrolene and nimodipine in combination.

Figure 3 illustrates the effect of a 30 min incubation with 50 *μ*M dantrolene (Figure 3(a)), 50 nM nimodipine (Figure 3(b)), and both drugs in combination (Figure 3(c)) on the ACh-induced relaxation in aortic rings from control rats. Endothelial-dependent relaxation and EC_50_ and *E*_max_ values were not affected by the drugs when used individually or in combination (Table 4). In diabetic rats, by contrast, endothelial function was significantly improved after incubation with dantrolene and with a combination of dantrolene and nimodipine. The *E*_max_ values for the ACh-induced relaxation increased from 56.46 ± 5.14% before treatment to 95.71 ± 6.15% after dantrolene and to 96.21 ± 7.50% after treatment with the dantrolene-nimodipine combination (*n* = 7, *P* < 0.05). Nevertheless, EC_50_ values remained unchanged (Figures 4(a) and 4(b), Table 4).

**Figure 5:**
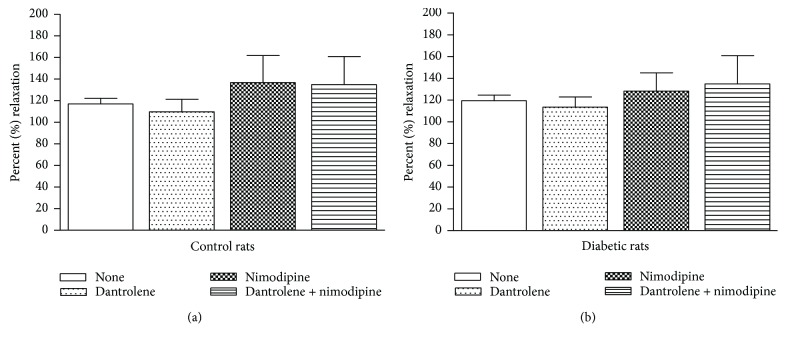
Effects of a 30 min incubation period with 50 *μ*M dantrolene, with 50 nM nimodipine, and with both drugs in combination on the endothelial-independent relaxation of aortic rings from control (a) and STZ diabetic rats (b). Aortic rings were precontracted with 0.1 *μ*M phenylephrine (PHE) before the addition of 1.0 *μ*m sodium nitroprusside (SNP) to directly relax the vascular smooth muscle. The values shown are the means ± SEM of 6 to 9 animals per group. Note that the drugs, either alone or in combination, did not modify endothelial-independent relaxation in control or STZ diabetic rats.

Figure 5 depicts the effects of a 30 min incubation period with 50 *μ*M dantrolene, 50 nM nimodipine, and both drugs in combination on the VSM relaxation elicited by 1.0 *μ*M SNP in aortic rings from control rats (Figure 5(a)) and from diabetic rats (Figure 5(b)). The addition of SNP to aortic rings that were precontracted with 0.1 *μ*M PHE produced similar endothelial-independent relaxation before and after incubation in both diabetics and controls, indicating that neither of these two drugs, either alone or in combination, modified the endothelial-independent relaxation in diabetic or control rats.

**Figure 6:**
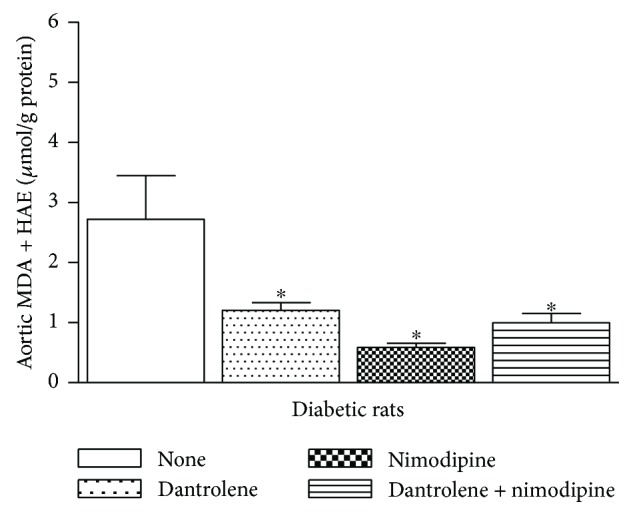
Effects of a 30 min incubation period with dantrolene (50 *μ*M), with nimodipine (50 nM), and with both drugs in combination on MDA + 4-HAE levels in aortic homogenates from STZ diabetic rats. Lipid peroxidation levels were significantly reduced by dantrolene, nimodipine, and both drugs in combination. The values shown are the means ± SEM of 5 animals per group. ^∗^*P* < 0.05, when comparing treated and untreated homogenates.

Figure 6 shows the effects of acute incubation with dantrolene, nimodipine, and both drugs in combination on MDA and 4-HAE levels in aortic homogenates from diabetic rats. MDA and 4-HAE levels (*μ*mol/g protein) were reduced from 2.72 ± 0.72 before incubation to 1.21 ± 0.13 with dantrolene, to 0.59 ± 0.07 with nimodipine, and to 1.00 ± 0.16 with both drugs in combination (*n* = 5, *P* < 0.05).

## 6. Discussion

We assessed the effects of dantrolene alone and in combination with nimodipine on both PHE-induced contraction and ACh-induced relaxation in the vasculature of type 1 diabetic rats. We found that the combination of dantrolene and nimodipine has synergistic effects in reducing the PHE-induced contraction in both diabetic and nondiabetic rats, but dantrolene alone or in combination with nimodipine improves ACh-dependent relaxation only in diabetic rats.

To our knowledge, this study is the first to report that dantrolene improves the endothelial-dependent relaxation under hyperglycemic conditions. This improvement in relaxation is fully blocked by L-NAME, suggesting that it is NO-dependent. That dantrolene reduces the lipid peroxidation markers MDA and 4-HAE indicates that the drug may also have antioxidant properties. Similarly, after traumatic spinal cord injury in rabbits, dantrolene treatment results in a significant decrease in MDA levels in cerebrospinal fluid and augments endogenous enzymatic and nonenzymatic antioxidative defenses [14]. Following intracranial hypertension in rats, by contrast, dantrolene alone fails to prevent the myocardial dysfunction resulting from oxidative stress [26].

Oxidative stress reduction by dantrolene may underlie improvements in endothelial-dependent relaxation in diabetic rats. This finding is relevant to the current study because oxidative stress, endothelial dysfunction, and augmented vascular tone are hallmarks of vascular deterioration in both diabetic animals and patients [7–9]. Indeed, following a four-week treatment period with statins, type 1 diabetic rats show an improvement in endothelial-dependent relaxation that is secondary to decreased oxidative stress [12, 23]. This reduced oxidative stress decreases vascular remodeling and improves cardiovascular function [12, 23]. Because preexisting diabetes mellitus is independently and strongly correlated with cerebral vasospasms in diabetic patients despite intensive glycemic control [27], dantrolene may provide a promising therapeutic tool. In addition to working synergistically with Ca^2+^ channel blockers to reduce agonist-induced VSM contraction, it may decrease vascular tone by improving endothelial-dependent relaxation. Despite improved ACh-induced relaxation, neither dantrolene nor its combination with nimodipine modifies SNP-induced relaxation in either diabetics or controls, indicating that these drugs do not alter the cGMP cascade in VSM.

Unlike dantrolene, nimodipine reduces MDA and 4-HAE levels in vascular homogenates, but it fails to improve vascular relaxation in diabetic rats. The latter finding differs from that of other studies with nondiabetic rats, which indicate that, by reducing oxidative stress, nimodipine enhances blood flow in cerebral ischemia [28]. Differences in experimental animal models, nimodipine doses, and treatment duration may partly explain these diverse findings.

At the tested dose, dantrolene alone does not reduce vascular PHE-induced contraction, but it does increase EC_50_ values in both diabetics and controls, making the vasculature less reactive to the alpha1-agonist. The mechanisms underlying this beneficial effect are unknown, although it has been proposed that the Ca^2+^ released from RyRs provides an increased pool of Ca^2+^ for the positive feedback on inositol 1,4,5-trisphosphate receptors [29]. Thus, blockade of RyRs with dantrolene may interrupt this feedback and reduce the response of agonist-induced inositol 1,4,5-trisphosphate receptors. Alterations in the kinetics of the PHE-alpha1 receptor interaction induced by dantrolene may also be responsible for the increased EC_50_ values, but specific binding studies are needed to verify this possibility.

Nimodipine significantly inhibits the PHE-induced contraction in both diabetics and controls, but the extent of the inhibition is less in diabetics (37%) than in controls (53%). The latter finding indicates that under hyperglycemic conditions, VSM regulation changes from that in controls. Similarly, the sensitivity of L-type Ca^2+^ channels to nifedipine is altered in diabetic rats, and the density of these channels is significantly less in VSM from diabetic rats than that from nondiabetic rats [2]. Thus, the modifications in Ca^2+^ homeostasis and vascular function, which result from abnormalities in L-type Ca^2+^ channels that are characteristics of diabetes, may explain, at least in part, the differences found in this study between diabetics and controls in the inhibition of the PHE-induced contraction with nimodipine.

In both diabetics and controls, the combination of dantrolene and nimodipine has synergistic effects that decrease the *E*_max_ of the PHE-induced contraction by approximately 80%. This synergistic effect has been previously reported in isolated basilar and femoral arteries from nondiabetic rats where the combination of these drugs significantly reduced 5-HT-induced vasoconstriction [22]. We found comparable results for diabetic rats. The mechanisms underlying the synergy between dantrolene and nimodipine in reducing the response of receptors linked to inositol 1,4,5-trisphosphate cascade are unknown. By increasing the EC_50_ value of the PHE-alpha1 receptor-mediated response, however, the combination of dantrolene and nimodipine may contribute to reducing vascular hyper-reactivity in diabetes, even though the vascular alpha1-mediated response is increased in this condition [30, 31].

## 7. Limitations of the Study

One limitation of this study is that the effects of dantrolene and nimodipine were only evaluated for rings from a systemic artery (thoracic aorta), rather than for rings from the cerebral vasculature. All three members of the RyR family (RyR1, RyR2, and RyR3), however, are present in the VSM of not only the thoracic aorta [15] but also the mesenteric arteries [15], the large cerebral arteries [16], and the cerebral microcirculation [5]. Therefore, the results of the current study may offer the first step in developing a customized therapy for reducing CVSPs in diabetics. To lend greater validity to our findings, however, future experiments are needed to evaluate the effects of dantrolene on the cerebral circulation, such as measuring blood flow velocities in the middle cerebral artery of diabetic rats.

## 8. Conclusions

We found that the combination of dantrolene and nimodipine has synergistic effects, which significantly decrease the PHE-induced contraction in both diabetic and control rats. Moreover, this combination reduces lipid peroxidation and improves vascular function in diabetic rats. Further studies are necessary, however, to prove that the response of aortic rings to these two drugs is similar in the cerebral vasculature. If a similar response does indeed occur, the combined use of dantrolene and nimodipine may be effective in reducing CVSPs in diabetics.

## Figures and Tables

**Table 1:** Blood glucose levels (mg/dl) of diabetic and control rats.

	Day 0	Day 1	Day 7	Day 14	Day 28
Control	166.3 ± 6.8	154.8 ± 6.6	127.8 ± 7.1	128.1 ± 4.2	143.5 ± 6.8
Diabetic	174.3 ± 13.1	451.7 ± 41.5^∗^	417.0 ± 59.9^∗^	446.4 ± 71.9^∗^	445.4 ± 63.7^∗^

Values are the means ± SEM. Rats were injected with STZ (65 mg/kg) on day 0. *n* = 20 rats per group. ^∗^*P* < 0.05, diabetics compared to age-matched controls.

**Table 2:** Body weight (g) of diabetic and control rats.

	Day 0	Day 1	Day 7	Day 14	Day 28
Control	180.22 ± 5.99	172.53 ± 5.24	282.3 ± 6.5	331.3 ± 4.9	360.6 ± 5.9
Diabetic	195.4 ± 3.3	199.5 ± 6.3	239.3 ± 14.3^∗^	254.9 ± 20.0^∗^	272.8 ± 24.8^∗^

Values are the means ± SEM. Rats were injected with streptozotocin on day 0. *n* = 20 rats per group. ^∗^*P* < 0.05, diabetics compared to age-matched controls.

**Table 3:** Effects of a 30 min incubation period with 50 *μ*M dantrolene (D), 50 nM nimodipine (N), and both drugs in combination (D + N) on EC_50_ and *E*_max_ values for PHE-induced contraction in control and diabetic rats.

Condition	*E*_max_ (g/mg tissue)	EC_50_ (nM)
Control none	0.41 ± 0.03	43.2 ± 8.11
Control + D	0.31 ± 0.03	118.72 ± 17.27^∗^
Control + N	0.19 ± 0.03^∗^	40.74 ± 10.20
Control D + N	0.08 ± 0.01^∗^	123.11 ± 34.15^∗^
Diabetic none	0.46 ± 0.04	50.83 ± 7.78
Diabetic + D	0.33 ± 0.08	118.73 ± 16.47^∗∗^
Diabetic + N	0.29 ± 0.04^∗∗^	48.71 ± 16.78
Diabetic D + N	0.08 ± 0.01^∗∗^	98.06 ± 34.35^∗∗^

Values shown are the means ± SEM of an average of 6 to 9 animals per group. ^∗^*P* < 0.05, when comparing treated and untreated aortic rings of controls. ^∗∗^*P* < 0.05, when comparing treated and untreated aortic rings of diabetics.

**Table 4:** Effects of a 30 min incubation period with 50 *μ*M dantrolene (D), 50 nM nimodipine (N), and both drugs in combination (D + N) on EC_50_ and *E*_max_ values for ACh-induced relaxation in control and diabetic rats.

Condition	*E*_max_ relaxation, %	EC_50_ (*μ*M)
Control none	80.66 ± 4.32	1.548 ± 1.197
Control + D	76.42 ± 8.48	0.337 ± 0.126
Control + N	72.87 ± 3.94	0.466 ± 0.176
Control D + N	87.27 ± 3.51	0.121 ± 0.003
Diabetic none	56.46 ± 5.14	0.490 ± 0.200
Diabetic + D	95.71 ± 6.15^∗∗^	0.465 ± 0.231
Diabetic + N	57.57 ± 6.30	0.950 ± 0.414
Diabetic D + N	96.21 ± 7.50^∗∗^	0.115 ± 0.090

Values shown are the means ± SEM of an average of 6 to 9 animals per group. ^∗∗^*P* < 0.05, when comparing treated and untreated aortic rings of diabetics.

## Abbreviations

ACh: Acetylcholine
CVSP: Cerebral vasospasms
EC_50_: Concentrations inducing 50% of maximal relaxation
*E*_max_: Maximal relaxation achieved
eNOS: Endothelial nitric oxide synthase
4-HAE: 4-Hydroxyalkenal
iNOS: Inducible nitric oxide synthase
L-NAME: N^G^-Nitro-L-arginine
MDA: Malondialdehyde
NO: Nitric oxide
PHE: Phenylephrine
RyR: Ryanodine receptor
SNP: Sodium nitroprusside
SR: Sarcoplasmic reticulum
STZ: Streptozotocin
VSM: Vascular smooth muscle.
